# Insecticidal Activity of 28 Essential Oils and a Commercial Product Containing *Cinnamomum cassia* Bark Essential Oil against *Sitophilus zeamais* Motschulsky

**DOI:** 10.3390/insects11080474

**Published:** 2020-07-27

**Authors:** Yunho Yang, Murray B. Isman, Jun-Hyung Tak

**Affiliations:** 1 Department of Agricultural Biotechnology, Seoul National University, Seoul 08826, Korea; uknowsheep@snu.ac.kr; 2 Faculty of Land and Food Systems, University of British Columbia, Vancouver, BC V6T 1Z4, Canada; murray.isman@ubc.ca; 3 Research Institute of Agriculture and Life Sciences, Seoul National University, Seoul 08826, Korea

**Keywords:** maize weevil, essential oil, cinnamon oil, fumigant toxicity, contact toxicity, attraction inhibition, efficacy, formulation

## Abstract

Maize weevils, *Sitophilus zeamais*, are stored product pests mostly found in warm and humid regions around the globe. In the present study, acute toxicity via contact and residual bioassay and fumigant bioassay of 28 essential oils as well as their attraction–inhibitory activity against the adults of *S. zeamais* were evaluated. Chemical composition of the essential oils was analyzed by gas chromatography-mass spectrometry, and a compound elimination assay was conducted on the four most active oils (cinnamon, tea tree, ylang ylang, and marjoram oils) to identify major active constituents. Amongst the oils examined, cinnamon oil was the most active in both contact/residual and fumigant bioassays, and exhibited strong behavioral inhibitory activity. Based on the compound elimination assay and chemical analyses, *trans*-cinnamaldehyde in cinnamon oil, and terpinen-4-ol in tea tree and marjoram oils were identified as the major active components. Although cinnamon oil seemed promising in the lab-scale bioassay without rice grains, it failed to exhibit strong insecticidal activity when the container was filled with rice. When a cinnamon oil-based product was applied both in an empty glass jar and a rice-filled container, all weevils in the empty jar were killed, whereas fewer than 15% died in the rice-filled container.

## 1. Introduction

Stored-product weevils including the maize weevil, *Sitophilus zeamais* Motschulsky, and the rice weevil, *S. oryzae* Linnaeus, belonging to the family Curculionidae, show cosmopolitan distribution, occurring in numerous warm and humid regions worldwide [1]. Maize weevils not only cause significant damage in stored grains including the reduction in nutritional quality, weight and germination rates of seeds in developing countries [2,3], but are also associated with human health due to allergen production and food safety in developed counties as well, since they can transmit fungi including *Aspergillus flavus* and several types of bacteria [4]. While the fumigant, methyl bromide was phasing-out and banned due to environmental issues, phosphine became the most frequently selected fumigant for stored pest control. However, heavy reliance on phosphine has resulted in the resistance development of resistance in numerous stored product insects including *Sitophilus* spp. in grain stores [5] as well as in the food industry and flour mills [6].

There is the considerable interest in screening and development of safer alternatives, and botanicals have been receiving great attention. With a few exceptions such as nicotine, botanicals tend to pose little threat to human health and the environment owing to their low mammalian toxicity and minimal environmental persistence [7]. Essential oils can be extracted from various plant parts including barks, flowers, buds, leaves, peels, and resins, mainly by a steam distillation method, and may contain hundreds of different monoterpenes, sesquiterpenes, and their derivatives. Essential oils are known to display various biological activities including acute and chronic toxicity, repellent activity, and inhibition of oviposition, growth, feeding and development against insect pest species [8,9,10].

The aim of this study was to evaluate the insecticidal activity of 28 essential oils and their attraction–inhibitory (i.e., deterrent) activity to rice grains using the adults of *S. zeamais* in laboratory bioassays. GC-MS analyses and compound elimination assays were performed to identify the major active constituents of the active oils, and the efficacy of a cinnamon oil-bearing commercial product was examined as well.

## 2. Materials and Methods

### 2.1. Test Insects

The maize weevils used in this study was originally collected from home storages in Yongin, South Korea (37°11′02.2″ N 127°12′24.8″ E) in late 2018, and the colony had been maintained in an insectary at Seoul National University, Seoul, South Korea, without exposure to any known insecticides at 26 ± 1 °C, 50–60% RH, and a 14:10 h L:D photoperiod. The colony was kept in a 2 L plastic container containing 800 g of rice grains (*Oryza sativa* L.). Unsexed (both male and female) adult weevils less than 1-month old were used in all experiments.

### 2.2. Test Essential Oils, Compounds and Commercial Product

The essential oils used in the present study are listed in Table 1. Bergamot, mandarin, and orange sweet oils were cold-pressed oils, and all the remaining oils were prepared via a steam distillation method from various plant parts including barks, flowers, buds, leaves, peels, and resins, which were purchased from Absolute Aromas (Hampshire, UK), Klimtech (Dimitrovgrad, Bulgaria), Plant Therapy (Twin Falls, ID, USA), or Sun Essential Oils (Phoenix, AZ, USA).

Pure chemical compounds in the essential oils were obtained in their technical grades, which were of the highest purity available. *o*-Cymene (>99.0%) and (e)-4-methoxycinnamaldehyde (>97.0%) were purchased from Tokyo Chemical Industry Co., Ltd. (Tokyo, Japan), and benzyl acetate (≥99%), benzyl benzoate (≥99%), benzyl salicylate (98%), β-caryophyllene (≥80%), *trans*-cinnamaldehyde (99%), cinnamyl acetate (99%), coumarin (≥99%), eucalyptol (99%), geranyl acetate (≥97%), linalool (97%), linalyl acetate (≥97%), methyl benzoate (99%), 4-methylanisole (99%), (+)-α-pinene (98%), (–)-terpinen-4-ol (≥95%), α-terpinene (≥89%), γ-terpinene (≥95%), α-terpineol (90%), and terpinolene (≥85%) were from Sigma-Aldrich (St. Louis, MO, USA). Two of the positive control insecticides, deltamethrin (>97%) and ethyl formate (97%), were purchased from Tokyo Chemical Industry Co., Ltd. and Sigma-Aldrich, respectively.

To evaluate the efficacy of a commercial product containing cinnamon oil as its active ingredient, ‘Rice Weevil Eradication’ (manufacturer: Hub Club, Siheung, Korea) was purchased from an online retail market (Auction, http://www.auction.co.kr/). The product was made with its liquid contents sealed in a breathable non-woven fabric, and evaporation was initiated when the seal was removed.

### 2.3. Chemical Composition of Essential Oils and Commercial Products

To identify the major constituents of the oils and test product, gas chromatography-mass spectrometry (GC-MS) analyses were performed with an ISQ gas chromatograph mass spectrometer (Thermo Scientific, Waltham, MA, USA) operating in EI mode fitted with a VF5ms column (60 m × 0.25 mm i.d., 0.25 µm thickness). Helium (99.999%) was used as a carrier gas with the constant flow of 1.0 mL/min. The injection volume was 1.0 µL, and the initial temperature for the oven was set at 50 °C for 5 min, then increased to 65, 120, 180, 210, and 325 °C with each rate of 10, 5, 5, 5, and 20 °C/min, respectively. Each stage was held for 30, 10, 0, 10, and 10 min, respectively. The data were analyzed using NIST Mass Spectral Search software (version 2.0), and the major constituents were determined by matching the spectra against the NIST/EPA/NIH Mass Spectral Libraries.

To monitor the changes in chemical composition of the commercial product during use, the seal of the product was opened and the liquid contents were allowed to evaporate at room temperature for 0, 1, and 2 months, respectively, and GC-MS analyses were conducted. At each time of monitoring, three aliquots were analyzed for a total of nine samples.

### 2.4. Bioassays

#### 2.4.1. Acute Toxicity of Plant Essential Oils

The insecticidal activity of the 28 oils was evaluated via a contact and residual application and a fumigation method described by Tak et al. [11] with a slight modification. For the contact and residual application assay, a dose of up to 150 mg of each essential oil in 200 μL of acetone was applied to a filter paper (Whatman No. 2, 5.5 cm in diameter) and allowed to dry for 2 min. The treated filter paper was placed into a Petri dish (Hyundai micro, Anseong, South Korea, 6.0 cm in diameter), and ten adults of the same age of *S. zeamais* were released into the Petri dish then sealed with Parafilm. Negative control had a filter paper treated with acetone alone, and the synthetic pyrethroid insecticide, deltamethrin, was used as a positive control. The dishes were held under the same conditions as mentioned above for colony maintenance.

For the fumigation assay, ten weevil adults were placed in a 1.5 mL micro centrifuge tube aerated with 200-mesh screen on both ends of the tube (Figure S1), and the tube was placed to the bottom of a 155 mL plastic cup. Up to 100 mg of each essential oil in 200 μL of acetone was applied to a filter paper and allowed to dry for 2 min, then placed in the cup with the lid closed. Negative control received acetone alone and ethyl formate was used as a positive control. Mortality was recorded after 24 h, with weevils considered dead if their appendages did not move when prodded with fine point forceps. All treatments were replicated at the minimum of three times (up to nine times) using different cohorts of weevils.

#### 2.4.2. Behavioral Attraction–Inhibitory Activity to Rice Grains via No-Choice Assay

**Figure 1:**
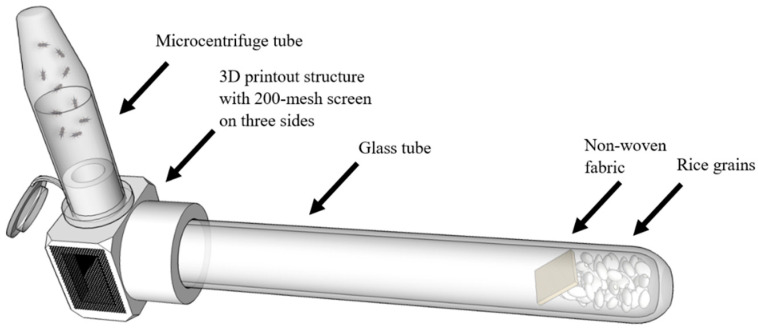
Schematic diagram of attraction–inhibitory activity assay.

In the attraction–inhibitory activity bioassay, 10 adult weevils which were starved for 24 h prior to the test and put into a 1.5-mL micro centrifuge tube. Approximately 1.3 g of rice grains was placed in the bottom of a borosilicate glass test tube (1.1 cm i.d. × 10 cm in length) with a piece of non-woven fabric (1 × 1 cm) located on top of the grains. Since the fabric piece was too small to hold a large volume of test solution, 50 μL of undiluted crude essential oil was directly applied to it using a micropipette; then, the glass tube was promptly assembled to a 3D printout structure (port), and laid horizontally (Figure 1). Ten weevils kept in the microcentrifuge tube were introduced through a hole in the port. The number of weevils that initiated attraction behavior to the glass tube containing rice grains was recorded at 1, 3, and 24 h post-treatment. The grains with non-treated fabric were used as the negative control, and the test was repeated three times. Percent inhibition (PI) for a given time point was calculated using the following formula [12];

PI (%) = (*N* − *n*)/*N* × 100

where *N* is the total number of insects introduced, and *n* is the number of insects attracted.

#### 2.4.3. Compound Elimination Assay

To examine the contribution of each major constituent of the essential oils to the overall contact and/or fumigant toxicity, a compound elimination assay was conducted on the four most active oils (cinnamon, tea tree, ylang ylang, and marjoram oils). Major compounds which constitute >2% of each oil were blended according to their natural ratio to make an artificial full mixture (FM), and a series of artificial mixtures was prepared by excluding each compound from FM [13]. The missing volume of excluded compound was supplemented with acetone, and the dose or concentration of each artificial mixture was prepared at the equivalent level of LD_95_ or LC_95_ of the original oils. The insecticidal activity of the artificial mixtures and corresponding oils was compared via either the contact or fumigation bioassay as mentioned above.

#### 2.4.4. Efficacy of a Commercial Product

The insecticidal activity of a commercial product was evaluated in two different test settings: a 13-L plastic container, and a 500-mL Mason jar. For the 13-L container test, rice grains were filled in six of 50-mL Conical tubes aerated with mesh screen on both ends, and 50 adult maize weevils were introduced into each tube. Three of the tubes were placed in the bottom of the container, and 10 kg of rice were filled, with the remaining three tubes buried on top of the grains. Test products which were either 0, 1 or 2 months-aged after the seal of the wrapper was opened in room condition were placed on the top of the rice and the lid was covered. The container was held at room temperature, and weevil mortality was recorded two weeks after treatment. The negative control did not contain the product, only the rice grains.

For the 500-mL container test, two 1.5-mL aerated micro centrifuge tubes containing 10 weevils were placed in the bottom and another two on top of the jar, and the container was either filled with rice or remained empty, and the newly sealed-off product was introduced into the container. The mortality was observed 24 h post-introduction of the products, and all tests were repeated three times.

### 2.5. Statistical Analysis

Probit analyses were conducted to determine LD_50_ or LC_50_ values of the essential oils and insecticides, and mortality in the compound elimination assay and attraction–inhibition assay were subjected to analysis of variance (ANOVA) using SPSS software (version 2.5, IBM, Armonk, NY, USA).

## 3. Results

### 3.1. Insecticidal Activity of Plant Essential Oils

The insecticidal activity of 28 essential oils against the adults of the *S. zeamais* are reported in Table 2. In the contact and residual bioassay, cinnamon oil was the most active oil (LD_50_ = 0.04 mg/cm^2^), followed by tea tree and marjoram oils (LD_50_ = 0.15 and 0.18 mg/cm^2^, respectively). Seven out of 28 oils failed to produce >50% of mortality at the highest dose tested (6.3 mg/cm^2^). Interestingly, several oils tested in the present study showed greater insecticidal activity than deltamethrin did, where the LD_50_ value of deltamethrin was 3.75 mg/cm^2^, indicating their strong residual effect, and presumably, complex insecticidal actions.

In the fumigation bioassay, we could evaluate LC_50_ values for only six essential oils (cinnamon, tea tree, ylang ylang, *E. radiata*, rosemary, *E. globulus* oils), since the remaining oils produced <50% mortality at the highest concentration tested. Cinnamon oil showed the greatest fumigant toxicity among the oils tested, followed by tea tree and ylang ylang oils (LC_50_ = 10.6, 25.1, and 52.0 mg/L air, respectively). Interestingly, some essential oils, including marjoram, peppermint, and Bulgarian lavender oils, which showed strong contact toxicity (LD_50_ < 0.31 mg/cm^2^) failed to exhibit corresponding fumigant toxicity effect (LC_50_ > 560.4 mg/L air). Several oils moderately active in the contact bioassay (0.36 < LD_50_ < 0.47 mg/cm^2^) also failed to produce notable fumigant toxicity effect, whereas some less toxic oils in the contact assay including *E. radiata*, rosemary, and *E. globulus* oils, had greater vapor toxicity than those mentioned above (LC_50_ values of 96.0, 121.8, and 137.9 mg/L air, respectively). Based on their LC_50_ values, cinnamon oil (10.6 mg/L air) showed greater toxicity than the positive control, ethyl formate (16.1 mg/L air).

### 3.2. Attraction–Inhibition via No-Choice Assay

**Figure 2:**
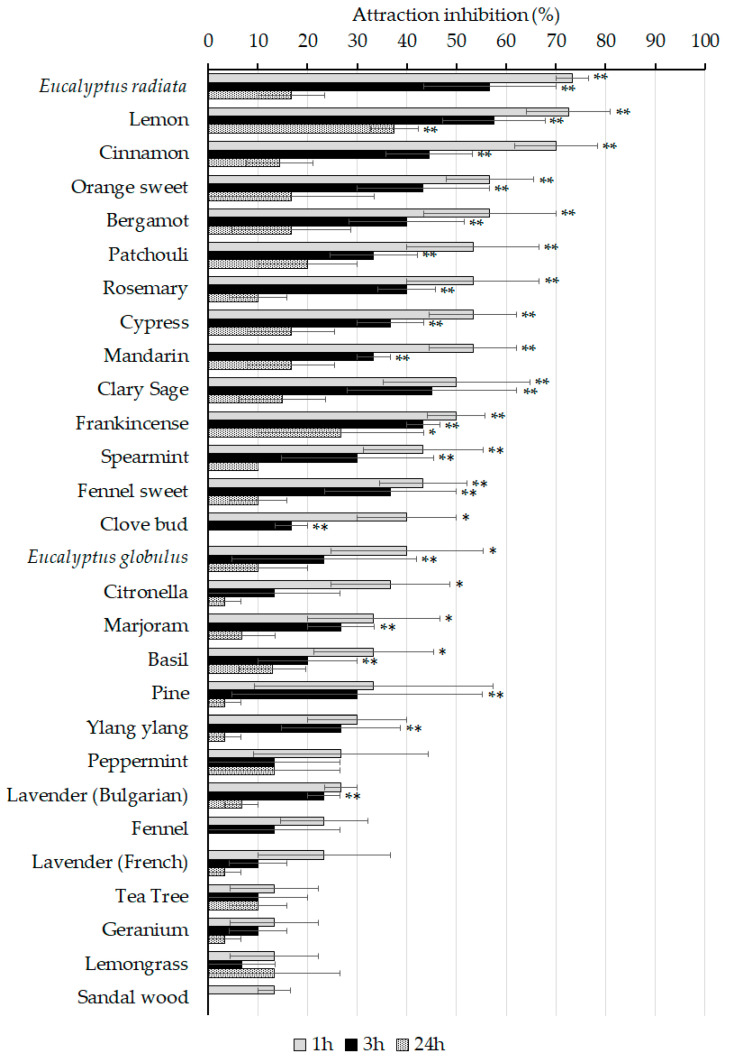
Attraction–inhibition activity. Control attraction–inhibition at 1, 3, and 24 h were 12.9 ± 4.3, 2.9 ± 1.7, and 7.1 ± 3.2%, respectively. Asterisks denote significant differences between the control repellency at *p* < 0.05 (*) and *p* < 0.01 (**) in one-way ANOVA followed by the Tukey’s b test.

The attraction–inhibitory activity of the 28 essential oils against the adults of *S. zeamais* was observed at 1, 3, and 24 h post-treatment (Figure 2). At 1 h after the release of the weevils, *E. radiata*, lemon, and cinnamon oils showed strong inhibition activity (>70%), and moderate activity (40–70% inhibition) was produced by 12 essential oils including mandarin, rosemary, patchouli, clary sage, frankincense, fennel sweet, bergamot, orang sweet, cypress, clove bud, *E. globulus*, and spearmint oils. Several oils active in the contact and residual toxicity bioassay, including tea tree, peppermint, Bulgarian lavender, and ylang ylang oils failed to generate notable attraction–inhibitory activity, showing no statistical difference to that of the control (*p* > 0.05).

Over time, the attraction–inhibition effect of the active oils diminished, possibly due to either the evaporation of the oils through the opening of the test chamber or the loss of concentration gradient in the air of test tubes. Whereas 18 and 20 oils showed significant inhibitory activity at 1 and 3 h post-treatment (*p* < 0.05), only frankincense and lemon oils displayed moderate activity (>40%) after 24 h of application.

### 3.3. Chemical Composition of Active Essential Oils

GC-MS analyses were conducted on all the essential oils tested, and the chemical compositions of the four most active oils in contact/residual and fumigation bioassays are listed in Table 3. The most abundant constituent in both the tea tree and marjoram oils was terpinen-4-ol (48.7 and 30.4%, respectively), and *trans*-cinnamaldehyde (74.6%) was the major constituent in cinnamon oil. Benzyl acetate (19.9%) was the most abundant constituent in ylang ylang oil, followed by linalool, benzyl salicylate, and 4-methylanisole, and their proportions in the oil were similar (18.0, 14.6, and 13.0% respectively). The full results of chemical analyses of 28 essential oils are available in Supplementary Materials (Tables S1–S28).

### 3.4. Comparative Toxicity of the Major Constituents

**Figure 3:**
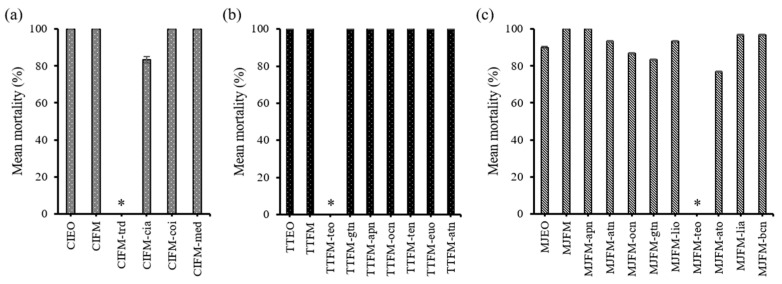
Compound elimination assay via contact and residual application: (**a**) cinnamon oil at LD_95_ of 0.22 mg/cm^2^; (**b**) tea tree oil at LD_95_ of 0.37 mg/cm^2^; (**c**) marjoram oil at LD_95_ of 0.37 mg/cm^2^ against *S. zeamais* adults. Asterisks denote significant differences at *p* = 0.05. (apn: (+)-α-pinene, atn: α-terpinene, ato: α-terpineol, bcn: β-caryophyllene, cia: cinnamyl acetate, coi: coumarin, euo: eucalyptol, gtn: γ-terpinene, lia: linalyl acetate, lio: linalool, med: (e)-4-methoxycinnamaldehyde, ocn: o-cymene, ten: terpinolene, teo: (–)-terpinen-4-ol, and trd: *trans*-cinnamaldehyde).

**Figure 4:**
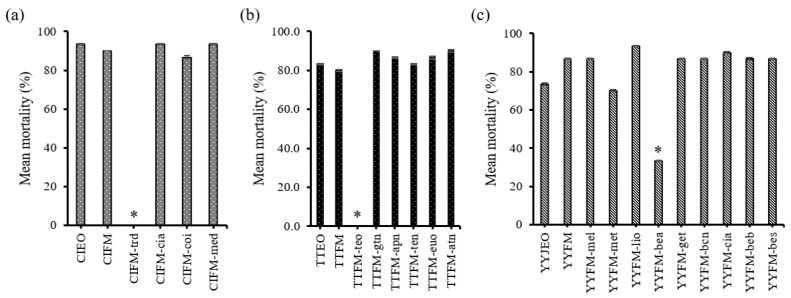
Compound elimination assay via fumigation assay: (**a**) cinnamon oil at LC_95_ of 238.6 mg/L air; (**b**) tea tree oil at LC_95_ of 114.5 mg/L air; (**c**) ylang ylang oil at LC_95_ of 142.0 mg/L air against *S. zeamais* adults. Asterisks denote significant differences at *p* = 0.05. (apn: (+)-α-pinene, atn: α-terpinene, bcn: β-caryophyllene, bea: benzyl acetate, beb: benzyl benzoate, bes: benzyl salicylate, cia: cinnamyl acetate, coi: coumarin, euo: eucalyptol, get: geranyl acetate, gtn: γ-terpinene, lio: linalool, med: (e)-4-methoxycinnamaldehyde, mel: 4-methylanisole, met: methyl benzoate, ten: terpinolene, teo: (–)-terpinen-4-ol, trd: *trans*-cinnamaldehyde).

In the compound elimination assay using marjoram and tea tree oils via the contact and residual application method, the artificial mixtures failed to cause any mortality to the adult weevils when terpinen-4-ol was excluded from the full mixtures (Figure 3a,b). On the other hand, the mortality of all the remaining combinations containing terpinen-4-ol including the full mixture showed no statistical difference when compared to those of the corresponding natural essential oils (*p* > 0.05), implicating terpinen-4-ol as the main constituent responsible for the insecticidal activity of those two oils (Figure 3b,c). Likewise, terpinen-4-ol was identified as the main active fumigant in tea tree oil, producing no mortality when removed from the full mixture (Figure 4b). Among the constituents of cinnamon oil, *trans*-cinnamaldehyde was shown to be the sole active compound in both the contact/residual and fumigant bioassays against the maize weevil, since no other compounds showed statistical difference when excluded from the full mixture (*p* > 0.05, Figure 3a and Figure 4a). Ylang ylang oil, although the artificial mixture lacking benzyl acetate, the most abundant compound, caused low mortality (<40%) that was statistically different (*p* < 0.05), it failed to completely nullify the toxicity unlike the other oils. Nonetheless, the other artificial mixtures showed no statistical difference in mortality when compared to that of the natural ylang ylang oil (*p* > 0.05, Figure 4c).

**Figure 5:**
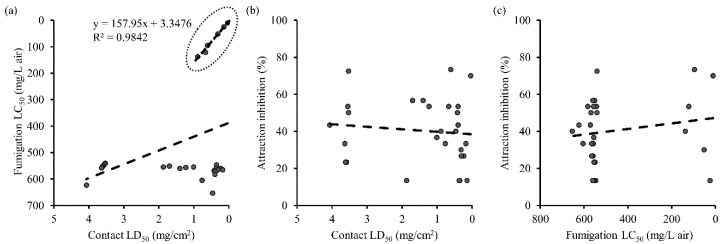
Correlation among contact/residual, fumigation, and anti-attraction activity of 28 essential oils tested: (**a**) contact/residual and fumigation (df = 1, 26; F = 3.60; *p* = 0.069); (**b**) contact/residual and 1 h attraction inhibition (df = 1, 26; F = 0.28; *p* = 0.600); (**c**) fumigation and 1 h attraction inhibition (df = 1, 26; F = 0.84; *p* = 0.367).

In the comparison between the values of LD_50_ in the contact and residual bioassay and LC_50_ in fumigant assay, six essential oils with strong contact toxicity were found to possess the equivalent level of fumigant toxicity, displaying high correlation between the two groups (*R*^2^ = 0.9842), whereas the other fifteen essential oils which showed contact toxicity (LD_50_ < 1.9 mg/cm^2^) failed to show corresponding fumigant toxicity (Figure 5). In the meantime, no direct correlation was found between attraction–inhibitory activity and either contact/residual or fumigant toxicity, with low *R*^2^ values of 0.011 and 0.031, respectively.

### 3.5. Chemical Composition and Efficacy of the Commercial Product

The label of the commercial product indicated cinnamon oil as its active ingredient, and GC-MS result confirmed the presence of *trans*-cinnamaldehyde in the product. While the newly opened product had 12.0% of this compound in its liquid contents, the concentration of the compound in the liquid increased to 53.1 ± 4.7% when the product remained open for two months, indicating its slower evaporation rate compared with other chemical constituents. Surprisingly, in terms of the efficacy of the product, it produced limited mortality for two weeks’ observation, with the greatest mortality at only 12.0 ± 6.7% in the one-month-old product (Table 4). It is notable that in the absence of rice, it showed complete mortality (100.0 ± 0.0%) within 24 h, whereas it failed to show any insecticidal activity when rice grains were present in the container, suggesting that rice grains counteract the efficacy of the oil or the product.

## 4. Discussion

Insecticide fumigation is one of the most widely adopted control methods for the protection of stored products from insect infestations. Plant-derived natural products are known to have relatively low mammalian toxicity, and they tend to be rapidly degraded in the environment, making them potential alternatives to conventional fumigants [14]. The insecticidal and repellent effect of plant extracts and essential oils against various stored product pests have been explored in many previous studies [15,16,17,18,19]. In this study, acute toxicity and attraction–inhibitory activity of 28 commercially obtained essential oils and their major constituents were examined against the adults of *S. zeamais*. Cinnamon oil showed the greatest contact and fumigant toxicity amongst the tested essential oils (Table 2). Cinnamon oil and *trans*-cinnamaldehyde, the most abundant constituent of the oil, are known to have insecticidal activity against several other coleopteran stored product insects including the rice weevil, *S. oryzae* L., Chinese bruchid, *Callosobruchus chinensis* L. [20], the red flour beetle, *Tribolium castaneum* Herbst [21], and the cigarette beetle, *Lasioderma serricorne* Fabricius [22]. The content of *trans*-cinnamaldehyde in the cinnamon oil in the present study was 74.6%, which was similar to that in *C. cassia* bark essential oil (66.3–77.2%) as reported by Li et al. [23]. According to Liu et al. [24], *trans*-cinnamaldehyde was identified as the major toxicant in *C. cassia* essential oil against the booklouse, *Liposcelis bostrychophila* Badonnel, and *trans*-cinnamaldehyde in *C. osmophloeum* essential oil also showed notable larvicidal activity on three species of mosquito larvae including the Asian tiger mosquito, *Aedes albopictus* Skuse, southern house mosquito, *Culex quinquefasciatus* Say, and *Armigeres subalbatus* Coquillett [25]. Our results from the compound elimination assay (Figure 3 and Figure 4) also revealed that *trans*-cinnamaldehyde acts as the major active compound for contact/residual and fumigation toxicity against the maize weevil. Besides cinnamon oil, marjoram and tea tree oils also showed highly effective contact toxicity, and terpinen-4-ol was the most abundant component in both oils. Terpinen-4-ol content in marjoram oil (30.4%) was comparable to the oils from other *Majorana hortensis* (*O. majorana*) plants in Egypt (30.0%) [26], whereas in our tea tree oil (48.7%) it was lower than oils of *M. alternifolia* plants in Brazil (53.7%) [27]. The difference in the proportion of the major constituents and the composition of minor constituents may vary depending on environmental [28] or nutritional factors [29]. Abbassy et al. [26] suggested that terpinen-4-ol is one of the main toxic constituents of marjoram oil against the black bean aphid, *Aphis fabae* Scop., and the Egyptian cotton leafworm, *Spodoptera littoralis* Boisduval. Likewise, compound elimination test results for marjoram and tea tree oils to *S. zeamais* in the present study indicated that terpinen-4-ol as the major contributing component. Seven other oils, including fennel, frankincense, lemon, mandarin, French lavender, pine and fennel sweet, did not appear to have any acute contact toxicity after 24 h of application (Table 2). As reported by Kim et al. [20], some methanol extracts from aromatic medicinal plant species that lacked acute toxicity against *S. oryzae* and *C. chinensis*, produced >90% mortality at 3 or 4 days post-treatment. Therefore, the possibility of prolonged insecticidal activity should not be ignored for plant extracts and/or essential oils even if their acute toxicity is unapparent.

In contrast to this study, Pavela et al. [30] reported that *F. vulgare* essential oil has an acute toxic effect on the larvae of *S. littoralis* and *C. quinquefasciatus* and the adults of *Musca domestica*, while this oil failed to show effective toxicity in the present study. Interspecific differences in biological activity, especially for insecticidal activity, are common and well-known in many insect pests. In earlier studies against four insect species (*S. littoralis* Fabricius, *Drosophila melanogaster* Meigen, *M. domestica*, *Diabrotica virgifera* LeConte) and the two-spotted spider mite, *Tetranychus urticae* Koch, toxicity of monoterpene compounds from essential oils including eugenol, carvacrol, α-terpineol, and terpinen-4-ol showed wide variety in their LD_50_ or LC_50_ values [31]. These types of discrepancy in toxicity can be intriguing in many fields of research, including biochemical research on detoxification, physicochemical study on cuticular penetration, electrophysiological studies of antennal perception of airborne particles, and physiological work on modes-of-action to elucidate the underlying mechanisms for interspecific difference in toxicity and repellent activity. In terms of different modes-of-action, these active essential oils seemed to possess different modes-of-action than deltamethrin. While deltamethrin displayed distinctive knock-down activity at relatively low dosages applied while many essential oils failed to exhibit any acute toxic responses, its LD_50_ value was greater than those of some active oils in the present study (Table 2). In the previous study of Fouad and da Camara [32], the LD_50_ for deltamethrin was 2.53 μL/mL against *S. zeamais* adults, which is equivalent to 0.03 mg/cm^2^, which is 140-fold more active than in our contact bioassay. One possible explanation for the significant difference in toxicity of the compound might be the different judgement standard of mortality, since we considered the weevils dead when they completely stopped moving when probed, regardless of their knock-down activity. Another possible reason is the difference in test methods, since we applied the compound onto filter papers whereas the previous study applied the insecticide directly on the glass surface of the Petri dish. Another previous study showed wide differences in the toxicity at the same dose of the same compounds but on different test surfaces [33].

Ylang ylang oil showed effective contact and fumigation toxicity, and GC-MS analysis data showed that 4-methylanisole, linalool, benzyl acetate, and benzyl salicylate were the major constituents comprising 13.0, 18.0, 19.9, and 14.6% of the oil, respectively. These monoterpenes and phenylpropanoid compounds are commonly found in the essential oils extracted from flowers of ylang ylang [34]. The compound elimination assay with ylang ylang oil demonstrated that the artificial full mixture excluding benzyl acetate (YYFM-bea in Figure 4c) showed lower mortality than the other combinations. Although the elimination of benzyl acetate from the full mixture resulted in a significant decrease in fumigant toxicity (*p* < 0.05), combination of the remaining constituents still exhibited modest insecticidal activity (33.3%), indicating that bioactivity of ylang ylang oil is cannot be solely attributed to benzyl acetate, but possibly in association with the remaining compounds, through either additive or synergistic interactions. Previous studies show complex interactions among the major constituents of essential oils against various insect and arthropod pests [35,36,37,38].

The attraction–inhibitory effect against adult *S. zeamais* decreased over time, as most of the oils showed no statistical difference in their 24-h activity when compared to the control except for frankincense and lemon oils (Figure 2). The inhibitory activity was most evident at 1 h post-treatment, with the average inhibition of 42.7%, and the most active treatment was *E. radiata* oil followed by lemon and cinnamon oils. Most constituents of plant essential oils are highly volatile due to their low molecular weight [39], and the volatility of essential oils can be affected by the types and structure of a test surface and formulation. For example, *E. radiata* oil was reported to have a repellent effect against *C. quinquefasciatus* for 8 h when applied on the skin of human volunteers [40]. Likewise, the repellent activity of 20 monoterpene compounds frequently found in many essential oils showed significant differences against two-spotted spider mites when applied to the leaves of bean and cabbage [33].

According to Obeng-Ofori et al. [41], the area preference test using 1,8-cineole, which is a major component of *Ocimum kenyense* oil, demonstrated strong repellent activity against *S. granarius* and *S. zeamais*, and the GC-MS result in this study indicated that the major component of *E. radiata* oil was eucalyptol (=1,8-cineole, 65.12%). Therefore, eucalyptol may have a major influence on the anti-attraction effect of *E. radiata* oil. The oils extracted from the fruit peels of plants belonging to the genus *Citrus* (lemon, orange sweet, bergamot, and mandarin) exhibited moderate attraction–inhibition against *S. zeamais* maintaining more than 50% activity at 1 h post-treatment. Peel oils of the genus *Citrus* are known to be rich in limonene [42], and our GC-MS analysis results (Tables S2, S16, S18 and S20) confirmed that limonene was the major component in bergamot (46.0%), lemon (75.7%), mandarin (71.9%), and sweet orange (83.8%) oils. A previous study reported the repellent and insecticidal activity of limonene [32], and the attraction–inhibition activity of those oils observed with *S. zeamais* could be attributed to limonene. Nonetheless, not all the acutely toxic essential oils induced strong inhibition on attraction to the rice grains. For instance, essential oils including tea tree and ylang ylang produced somewhat notable toxicity, but their inhibitory effect at 1 h post-treatment was not proportionate. In an earlier study, similar results were obtained by Tak and Isman [33] in that camphor, geranic acid, menthone, and α-pinene showed relatively strong or moderate toxicity against *Tetranychus urticae* but did not show a corresponding repellent effect. This indicates that toxicity cannot be directly related to the repellent or attraction–inhibitory effect, and complex and various mechanisms of action may be involved.

As the use of methyl bromide was being phased-out in the stored product pests control programs, phosphine fumigation became the most popular control method around the globe. Compared to other potential alternatives such as sulfuryl fluoride, carbonyl sulfide, propylene oxide, ethyl formate, and hydrogen cyanide, phosphine has unique benefits including lowest costs, various formulations that are easy to apply, rapid dispersion into the treated areas due to its similar density to air, and fast break down after fumigation [5]. However, the lack of compatible alternatives and repeated use of phosphine treatment in industrial storages as well as in flour mills has resulted in the development of resistance in various stored product insect pests, which emphasizes the need for additional pest management products. The current situation of phosphine resistance is well documented [5].

In household environments, on the other hand, the control of grain pests should not rely on synthetic chemical control due to safety concerns, since the grains are readily eaten by consumers in their homes. Botanical sources are frequently adopted as good alternatives in this particular situation, and many commercial products are available, especially in Asian countries including South Korea and Japan. Many of the products tend to be made from strong scented plants such as pepper, wasabi, and horseradish. The product tested in the present study used cinnamon oil as the active ingredient and, as shown in Table 2, the oil itself seems promising to control maize weevils. However, our experiment with the formulated product produced a result opposite to that expected when it was deployed with rice grains. In this case, the insecticidal activity was almost nullified by the grains. Possible explanations include the rapid absorption to the surface of rice grains, degradation of active compounds by the metabolic process of grains, or limited (or blocked) evaporation of active constituents. In a previous study, Lee et al. [43] observed a similar result in that fumigant toxicity of six essential oils against *S. oryzae* was three to nine times lower with a 50% filling ratio of wheat, compared to vessels lacking wheat. Likewise, the presence or absence of grain also significantly affected the fumigant toxicity of ethyl formate to *S. oryzae* as well [44]. Further studies should focus on understanding the absorption nature of essential oils and their active principles, and/or formulation approaches to control or decrease the absorption or attachment to the surface of grains to enhance the efficacy of control agents. Finally, organoleptic evaluation of the treated grains (e.g., for color, flavor, odor, taste, and texture) must be considered.

## 5. Conclusions

The present study evaluated the susceptibility of *S. zeamais* adults to the acute contact and vapor phase toxicity of 28 essential oils and their major compounds. Amongst 28 oils tested, cinnamon oil exhibited the greatest toxicity and attraction–inhibitory (=deterrent) activity to rice. Toxicity and attraction–inhibition activity were not correlated in most cases. *E. radiata* and cinnamon essential oils were the most active attraction inhibitors at 1 h, and several limonene-containing essential oils showed moderate activity in this bioassay. Although cinnamon oil was the most active in all three laboratory bioassays, a cinnamon oil-bearing commercial product showed limited efficacy with a rice-filled container. Further study is required to understand the absorption of active fumigants by grains, and to enhance efficacy through the formulation for household products.

## Figures and Tables

**Table 1:** Plant species and essential oils tested in this study.

Essential Oil	Family	Scientific Name	Plant Parts Extracted from	Manufacturer
Basil	Lamiaceae	*Ocimum basilicum*	leaf, flower	Sun Essential Oils
Bergamot	Rutaceae	*Citrus bigaradia*	peel	Klimtech
Cinnamon	Lauraceae	*Cinnamomum cassia*	bark	Plant Therapy
Citronella	Poaceae	*Cymbopogon nardus*	leaf	Absolute Aromas
Clary Sage	Lamiaceae	*Salvia sclarea*	flower	Klimtech
Clove bud	Myrtaceae	*Syzygium aromaticum*	flower bud	Absolute Aromas
Cypress	Cupressaceae	*Cupressus sempervirens*	leaf	Klimtech
*Eucalyptus globulus*	Myrtaceae	*Eucalyptus globulus*	leaf	Klimtech
*Eucalyptus radiata*	Myrtaceae	*Eucalyptus radiata*	leaf	Klimtech
Fennel	Apiaceae	*Foeniculum vulgare*	seed	Sun Essential Oils
Fennel sweet	Apiaceae	*Foeniculum vulgare*	seed	Klimtech
Frankincense	Burseraceae	*Boswellia carterii*	resin	Klimtech
Geranium	Geraniaceae	*Pelargonium graveolens*	flower	Klimtech
Lavender (French)	Lamiaceae	*Lavandula angustifolia*	flower bud	Absolute Aromas
Lavender (Bulgarian)	Lamiaceae	*Lavandula angustifolia*	flower	Klimtech
Lemon	Rutaceae	*Citrus limonum*	peel	Klimtech
Lemongrass	Poaceae	*Cymbopogon citratus*	leaf	Klimtech
Mandarin	Rutaceae	*Citrus reticulata*	peel	Klimtech
Marjoram	Lamiaceae	*Origanum majorana*	leaf	Klimtech
Orange sweet	Rutaceae	*Citrus aurantium*	peel	Klimtech
Patchouli	Lamiaceae	*Pogostemon cablin*	leaf	Klimtech
Peppermint	Lamiaceae	*Mentha piperita*	leaf	Klimtech
Pine	Pinaceae	*Pinus* spp.	needle	Sun Essential Oils
Rosemary	Lamiaceae	*Rosmarinus officinalis*	leaf	Klimtech
Sandal wood	Santalaceae	*Santalum album*	wood	Klimtech
Spearmint	Lamiaceae	*Mentha spicata*	leaf, flower	Absolute Aromas
Tea Tree	Myrtaceae	*Melaleuca alternifolia*	leaf	Klimtech
Ylang ylang	Annonaceae	*Cananga odorata*	flower	Klimtech

**Table 2:** Insecticidal activity of 28 essential oils against *Sitophilus zeamais* adults.

Essential Oils	Contact Toxicity			Fumigation Toxicity		
	LD_50_ (95% CL) *^a^*	Slope ± SE *^b^*	χ^2^ (d.f)	LC_50_ (95% CL) *^c^*	Slope ± SE	χ^2^ (d.f)
Cinnamon	0.04 (0.03–0.04)	7.3 ± 1.2	5.0 (16)	14.0 (11.6–16.8)	2.4 ± 0.3	32.0 (25)
Tea Tree	0.15 (0.14–0.16)	13.8 ± 2.1	5.7 (13)	18.3 (15.5–21.8)	4.9 ± 0.6	50.5 (22)
Marjoram	0.18 (0.15–0.23)	9.2 ± 1.3	94.2 (16)	>565.8	-	
Peppermint	0.24 (0.22–0.26)	8.1 ± 1.4	13.6 (13)	>560.4	-	
Lavender (Bulgarian)	0.31 (0.24–0.39)	2.5 ± 0.4	7.8 (10)	>565.2	-	
Ylang ylang	0.32 (0.23–0.43)	4.5 ± 0.7	22.1 (10)	52.0 (45.8–58.1)	3.8 ± 0.4	26.2 (31)
Geranium	0.36 (0.28–0.49)	1.8 ± 0.2	15.2 (16)	>547.3	-	
Lemongrass	0.37 (0.25–0.50)	1.5 ± 0.2	16.9 (19)	>560.2	-	
Patchouli	0.40 (0.35–0.49)	11.8 ± 1.9	45.8 (13)	>571.6	-	
Spearmint	0.40 (0.30–0.51)	1.5 ± 0.1	52.8 (40)	>583.2	-	
Clary Sage	0.42 (0.28–0.60)	1.3 ± 0.2	22.0 (19)	>568.8	-	
Clove bud	0.47 (0.35–0.60)	1.3 ± 0.1	31.2 (37)	>654.0	-	
*E. radiata*	0.61 (0.56–0.66)	6.5 ± 0.8	20.3 (25)	96.0 (75.7–121.1)	6.3 ± 0.8	198.1 (28)
Rosemary	0.67 (0.57–0.78)	3.7 ± 0.7	18.1 (13)	121.8 (107.8–133.0)	4.9 ± 0.6	29.9 (34)
Basil	0.77 (0.49–1.16)	1.5 ± 0.2	29.0 (19)	>605.2	-	
*E. globulus*	0.89 (0.82–0.96)	9.7 ± 1.5	9.4 (16)	137.9 (121.6–181.1)	8.9 ± 1.2	117.7 (22)
Citronella	1.01 (0.73–1.38)	1.3 ± 0.2	40.1 (31)	>555.5	-	
Cypress	1.23 (1.04–1.50)	4.5 ± 0.6	28.1 (16)	>556.8	-	
Orange sweet	1.40 (1.15–1.71)	2.6 ± 0.3	17.1 (16)	>560.2	-	
Bergamot	1.70 (1.05–2.66)	1.8 ± 0.3	23.2 (13)	>551.4	-	
Sandal wood	1.87 (1.49–2.39)	2.0 ± 0.3	22.0 (22)	>555.2	-	
Fennel	>3.59	–		>551.0	-	
Frankincense	>3.53	–		>541.6	-	
Lemon	>3.53	–		>541.1	-	
Mandarin	>3.55	–		>543.7	-	
Lavender (French)	>3.62	–		>554.6	-	
Pine	>3.64	–		>558.3	-	
Fennel sweet	>4.07	–		>623.4	-	
*trans*-Cinnamaldehyde	0.02 (0.02–0.02)	8.4 ± 1.3	11.5 (19)	12.1 (9.7–15.6)	2.8 ± 0.2	117.1 (40)
Terpinen-4-ol	0.06 (0.05–0.06)	15.3 ± 2.6	29.8 (19)	11.2 (10.2–12.2)	4.2 ± 0.4	41.2 (37)
Deltamethrin	3.75 (2.24–8.37)	1.0 ± 0.1	50.6 (40)	n.t. *^d^*		
Ethyl formate	n.t.			16.1 (13.8–18.9)	3.9 ± 0.4	92.5 (42)

*^a^* mg/cm^2^; *^b^* Standard Error; *^c^* mg/L air; *^d^* Not tested.

**Table 3:** Chemical constituents of four most active essential oils.

RT	Compounds	Tea Tree	Marjoram	Cinnamon	Ylang Ylang
27.22	α-Pinene	4.6	1.4	-	-
34.38	Sabinene	-	2.6	-	-
35.48	β-Pinene	-	1.0	-	-
40.73	3-Carene	-	2.7	-	-
41.08	4-Methylanisole	-	-	-	13.0
41.71	α-Terpinene	3.7	1.8	-	-
42.62	o-Cymene	5.6	9.0	-	-
43.03	Limonene	1.2	-	-	-
43.35	Eucalyptol	4.8	-	-	-
45.61	γ-Terpinene	15.8	6.1	-	-
47.54	Methyl benzoate	-	-	-	5.8
47.57	Terpinolene	6.8	2.2	-	-
48.52	Linalool	-	6.9	-	18.0
52.35	Benzyl acetate	-	-	-	19.9
54.84	Terpinen-4-ol	48.7	30.4	-	-
55.96	α-Terpineol	-	4.9	-	-
60.09	Linalyl acetate	-	11.2	-	-
60.88	Piperitone	-	1.3	-	-
62.51	*trans*-Cinnamaldehyde	-	-	74.6	-
66.78	Geranyl acetate	-	-	-	6.2
68.96	Undecanoic acid, methyl ester	1.7	1.9	1.8	3.0
69.36	β-Caryophyllene	-	8.5	-	4.8
69.58	Cinnamyl acetate	-	-	-	3.7
70.06	*trans*-Cinnamyl acetate	-	-	2.8	-
70.17	Coumarin	-	-	1.9	-
70.29	α-Caryophyllene	-	-	-	1.6
73.06	3-Methoxycinnamaldehyde	-	-	9.5	-
79.60	Benzyl benzoate	-	-	-	3.8
83.34	Benzyl salicylate	-	-	-	14.6
	total	92.9	91.8	90.6	94.4

**Table 4:** Insecticidal activity of a commercial product on the adult of the maize weevil.

Months Evaporated	Mortality (% ± SE)				*trans*-Cinnamaldehyde Content (% ± SE)
0	1.3 ± 0.8	2.1 ± 0.9	0.0 ± 0.0	100.0 ± 0.0 *	12.0 ± 0.0
1	1.0 ± 0.7	12.0 ± 6.7 *	-	-	23.4 ± 1.6
2	1.3 ± 0.7	1.8 ± 0.2	-	-	53.1 ± 4.7
Product	without	with	with	with	
Container volume	13 L	13 L	500 mL	500 mL	
Rice grain	with	with	with	without	

* Asterisks denote significant differences at *p* = 0.05.

## Supplementary Materials

The following are available online at https://www.mdpi.com/2075-4450/11/8/474/s1, Figure S1: Schematic diagram of fumigation assay arena, Tables S1–S28: Chemical constituents of each essential oils.
