# Supplementary material for: Insecticidal Activity of 28 Essential Oils and a Commercial Product Containing Cinnamomum cassia Bark Essential Oil against Sitophilus zeamais Motschulsky
Source: Insects. 2020 Jul 27;11(8):474. doi: 10.3390/insects11080474 (PMC7469181; doi:10.3390/insects11080474)
Supplement: Supplementary file 1 [file insects-11-00474-s001.pdf]

[Supplementary Information]

Insecticidal activity of 28 essential oils and a commercial product containing *Cinnamomum cassia* bark essential oil against *Sitophilus zeamais* Motschulsky

Yunho Yang[1], Murray B. Isman[2], and Jun-Hyung Tak[1,3]*

[1]Department of Agricultural Biotechnology, Seoul National University, Seoul 08826, Korea; uknowsheep@snu.ac.kr

[2]Faculty of Land and Food Systems, University of British Columbia, Vancouver, BC V6T 1Z4, Canada; murray.isman@ubc.ca

[3]Research Institute of Agriculture and Life Sciences, Seoul National University, Seoul 08826, Korea

*Correspondence: jhtak@snu.ac.kr; Tel.: +82-2-880-4702; fax: +82-2-873-2319

Table S1. Chemical constituents of basil essential oil

retention time (min)	constituent	area %
48.34	Linalool	19.7
54.08	Levomenthol	0.5
55.83	Estragole	70.3
59.18	Z-Citral	0.4
61.29	E-Citral	0.6
68.98	Undecanoic acid, methyl ester	1.7
69.27	β-Caryophyllene	0.5
69.61	*trans*-α-Bergamotene	0.7
71.50	Germacrene D	0.4
73.17	cis-α-Bisabolene	1.8
total		96.6

Table S2. Chemical constituents of bergamot essential oil

retention time (min)	constituent	area %
25.83	α-Thujene	0.5
27.00	α-Pinene	24.5
29.64	Camphene	0.7
34.88	β-Pinene	0.8
37.50	α-Myrcene	0.5
40.42	3-Carene	5.0
41.19	Isocineole	2.0
42.27	o-Cymene	0.4
42.80	D-Limonene	46.0
43.05	Eucalyptol	1.5
45.41	γ-Terpinene	0.4
47.43	α-Terpinolene	3.7
48.34	Linalool	3.3
55.54	α-Terpineol	0.7
60.04	Linalyl acetate	3.2
65.26	Triacetin	0.7
65.83	alpha-Terpinyl acetate	0.7
68.98	Undecanoic acid, methyl ester	2.1
total		96.6

Table S3. Chemical constituents of cinnamon essential oil

retention time (min)	constituent	area %
49.53	Phenylethyl Alcohol	0.3
59.80	2,3-Dihydro-benzofuran-3-ol	0.4
62.51	*trans*-Cinnamaldehyde	74.6
68.95	Undecanoic acid, methyl ester	1.8
70.06	*trans*-Cinnamyl acetate	2.8
70.17	Coumarin	1.9
73.06	3-Methoxycinnamaldehyde	9.5
76.69	1,5-Dihydroxy-1,2,3,4-tetrahydronaphthalene	0.3
total		91.7

Table S4. Chemical constituents of citronella essential oil

retention time (min)	constituent	area %
42.55	Limonene	3.8
48.20	Linalool	0.8
51.67	Isopulegol	1.2
51.98	Citronellal	35.7
52.47	Isopulegol	0.6
58.03	α-Citronellol	13.4
59.99	Geraniol	19.6
61.14	E-Citral	0.5
65.68	Citronellyl acetate	4.2
65.94	Eugenol	0.6
67.00	Geranyl acetate	2.6
67.78	α-elemene	1.7
68.87	Undecanoic acid, methyl ester	2.9
71.40	Germacrene D	1.2
71.89	α-Muurolene	0.7
72.42	γ-Muurolene	0.6
72.53	ë-Cadinene	2.2
73.45	Elemol	2.2
74.40	Cubenol	0.5
76.24	.tau.-Muurolol	0.4
76.57	α-Cadinol	0.5
76.67	α-Eudesmol	0.6
total		96.5

Table S5. Chemical constituents of clary sage essential oil

retention time (min)	constituent	area %
48.37	Linalool	32.4
55.54	α-Terpineol	3.9
55.97	2-Carene	0.4
58.06	Geraniol	1.4
60.07	Linalyl acetate	45.2
61.33	dihydro linalool	0.5
65.83	α-Terpinyl acetate	3.5
65.93	Ocimenyl acetate	0.3
66.24	Neryl acetate	3.1
67.12	Geranyl acetate	4.4
68.98	Undecanoic acid, methyl ester	2.0
total		97.2

Table S6. Chemical constituents of clove bud essential oil

retention time (min)	constituent	area %
65.91	Eugenol	94.4
68.69	Undecanoic acid, methyl ester	1.3
68.96	β-Caryophyllene	1.5
72.04	Phenol, 2-methoxy-4-(2-propenyl)-,acetate	2.1
total		99.3

Table S7. Chemical constituents of cypress essential oil

retention time (min)	constituent	area %
26.96	α-Pinene	1.7
29.62	Camphene	0.5
33.90	Sabinene	0.6
34.91	β-Pinene	48.8
37.47	α-Myrcene	2.7
40.41	3-carene	20.9
42.26	o-Cymene	1.0
42.76	Limonene	3.9
42.88	α-Phellandrene	0.4
47.42	α-Terpinolene	3.5
47.69	o-Isopropenyltoluene	0.4
48.33	Linalool	0.5
51.32	Isopinocarveol	0.4
54.37	Terpinen-4-ol	3.7
54.80	p-Cymen-8-ol	0.4
55.70	Myrtenol	0.4
65.83	α-Terpinyl acetate	3.9
65.94	Bicyclo[3.1.0]hexene, 6-isopropylo	0.4
68.98	Undecanoic acid, methyl ester	2.0
total		95.8

Table S8. Chemical constituents of *Eucalyptus globulus* essential oil

retention time (min)	constituent	area %
26.95	α-Pinene	2.3
40.39	3-Carene	1.9
42.25	o-Cymene	2.4
42.76	D-Limonene	5.7
43.06	Eucalyptol	75.6
45.39	γ-Terpinene	2.8
47.41	α-Terpinene	1.3
54.37	Terpinen-4-ol	0.3
67.36	α-Copaene	0.4
68.97	Undecanoic acid, methyl ester	1.8
69.27	β-Caryophyllene	0.8
total		95.3

Table S9. Chemical constituents of *Eucalyptus radiata* essential oil

retention time (min)	constituent	area %
26.97	α-Pinene	2.4
34.88	β-Pinene	0.5
37.49	α-Myrcene	0.3
40.41	3-carene	1.9
42.26	o-Cymene	2.8
42.77	D-Limonene	6.2
42.90	α-Phellandrene	0.4
43.07	Eucalyptol	65.1
45.40	γ-Terpinene	2.7
47.42	α-Terpinolene	1.3
54.37	Terpinen-4-ol	0.4
55.53	α-Terpineol	7.3
55.97	γ-Terpineol	1.1
67.36	α-Copaene	0.4
68.98	Undecanoic acid, methyl ester	1.8
69.27	β-Caryophyllene	0.9
total		95.5

Table S10. Chemical constituents of fennel essential oil

retention time (min)	constituent	area %
26.09	α-Pinene	3.2
36.32	α-Myrcene	0.7
39.22	l-Phellandrene	1.9
42.14	D-Limonene	2.6
47.17	Fenchone	1.2
62.12	Anethole	44.6
68.66	Undecanoic acid, methyl ester	21.2
69.09	Undecanoic acid, 2-methyl-	1.3
total		76.7

Table S11. Chemical constituents of fennel sweet essential oil

retention time (min)	constituent	area %
26.08	α-Pinene	0.8
41.60	o-Cymene	1.0
42.14	D-Limonene	4.0
47.15	L-Fenchone	1.5
55.19	Estragole	3.6
59.95	Anisaldehyde	2.1
62.28	Anethole	79.6
68.65	Undecanoic acid, methyl ester	3.2
total		95.8

Table S12. Chemical constituents of frankincense essential oil

retention time (min)	constituent	area %
25.08	2-Thujene	15.3
26.27	α-Pinene	44.4
32.82	Sabinene	7.5
33.71	β-Pinene	2.7
36.33	α-Myrcene	1.4
39.64	3-Carene	1.0
41.66	o-Cymene	6.1
42.22	Limonene	14.9
68.67	Undecanoic acid, methyl ester	2.8
total		96.3

Table S13. Chemical constituents of geranium essential oil

retention time (min)	constituent	area %
24.73	Hexylene glycol	0.4
48.36	Linalool	11.3
52.44	l-Menthone	0.9
53.16	p-Menthone	5.2
58.22	α-Citronellol	39.6
60.09	Geraniol	15.3
61.65	Citronellyl formate	11.0
63.19	Geraniol formate	4.7
65.82	Citronellyl acetate	0.8
67.13	Geranyl acetate	2.2
68.39	Diphenyl ether	0.6
68.78	α-Gurjunene	0.5
69.00	Undecanoic acid, methyl ester	2.2
69.59	Diphenylmethane	0.3
total		95.2

Table S14. Chemical constituents of lavender (French) essential oil

retention time (min)	constituent	area %
35.23	3-Octanone	0.8
42.94	*trans*-α-Ocimene	0.9
43.93	β-Ocimene	0.6
48.05	Linalool	33.6
48.48	1-Octen-3-yl-acetate	0.8
51.27	(-)-Camphor	0.5
59.64	Linalyl acetate	47.5
68.67	Undecanoic acid, methyl ester	3.7
68.77	α-Santalene	0.7
68.94	β-Caryophyllene	3.3
69.86	α-Farnesene	1.7
74.48	Caryophyllene oxide	0.6
total		94.7

Table S15. Chemical constituents of lavender (Bulgarian) essential oil

retention time (min)	constituent	area %
27.03	α-Pinene	0.3
42.31	o-Cymene	3.2
42.80	Limonene	2.2
43.07	Eucalyptol	3.5
48.39	Linalool	33.3
53.52	3,5,5-Trimethylhexyl acetate	0.4
54.41	Terpinen-4-ol	3.7
55.58	α-Terpineol	0.7
60.09	Linalyl acetate	35.5
61.36	Dihydro linalool	0.4
65.85	α-Terpinyl acetate	0.6
66.26	Neryl acetate	1.1
67.14	Geranyl acetate	3.4
69.00	Undecanoic acid, methyl ester	1.9
69.30	β-Caryophyllene	4.7
74.78	Caryophyllene oxide	1.2
total		96.1

Table S16. Chemical constituents of lemon essential oil

retention time (min)	constituent	area %
26.32	α-Pinene	3.1
34.01	β-Pinene	9.2
36.60	α-Myrcene	0.8
39.82	3-Carene	2.1
41.81	o-Cymene	1.2
42.53	D-Limonene	75.7
45.06	γ-Terpinene	4.5
47.11	α-Terpinolene	0.4
68.75	Undecanoic acid, methyl ester	0.8
total		97.7

Table S17. Chemical constituents of lemongrass essential oil

retention time (min)	constituent	area %
27.02	α-Pinene	1.1
42.80	Limonene	6.6
43.07	Eucalyptol	0.9
48.36	Linalool	3.5
54.23	Verbenol	0.4
58.20	α-Citronellol	12.5
59.24	Z-Citral	23.0
60.09	Geraniol	9.4
61.34	E-Citral	28.6
66.26	Neryl acetate	1.1
67.14	Geranyl acetate	3.7
69.00	Undecanoic acid, methyl ester	2.1
69.30	β-Caryophyllene	2.8
total		95.5

Table S18. Chemical constituents of mandarin essential oil

retention time (min)	constituent	area %
26.12	α-Pinene	2.7
33.71	β-Pinene	7.0
36.33	α-Myrcene	1.1
41.65	o-Cymene	2.6
42.34	D-Limonene	71.9
44.94	γ-Terpinene	6.2
68.66	Undecanoic acid, methyl ester	3.4
total		94.9

Table S19. Chemical constituents of marjoram essential oil

retention time (min)	constituent	area %
27.22	α-Pinene	1.4
34.38	Sabinene	2.6
35.48	β-Pinene	1.0
40.73	3-Carene	2.7
41.71	α-Terpinene	1.8
42.62	o-Cymene	9.0
43.03	Limonene	1.0
43.20	α-Phellandrene	0.5
45.61	γ-Terpinene	6.1
47.57	Terpinolene	2.2
48.52	Linalool	6.9
54.84	Terpinen-4-ol	30.4
54.99	α-Thujone	0.7
55.96	α-Terpineol	4.9
56.34	γ-Terpineol	0.5
60.09	Linalyl acetate	11.2
60.88	Piperitone	1.3
68.96	Undecanoic acid, methyl ester	1.9
69.36	β-Caryophyllene	8.5
total		94.6

Table S20. Chemical constituents of orange sweet essential oil

retention time (min)	constituent	area %
26.99	*α*-Pinene	0.4
37.52	*α*-Myrcene	0.4
42.83	Limonene	83.8
48.35	Linalool	0.3
49.89	*trans*-*p*-Mentha-2,8-dienol	0.6
50.71	Limonene oxide	0.4
50.94	*cis*-*p*-Mentha-2,8-dien-1-ol	0.6
51.02	*trans*-Limonene oxide	0.7
56.02	Perilla alcohol	0.5
57.65	*trans*-Carveol	1.1
58.78	*cis*-Carveol	0.5
59.20	Z-Citral	0.6
59.74	Carvone	1.3
61.31	E-Citral	0.8
65.69	7-Oxabicyclo[4.1.0]heptane	1.8
68.42	*cis*-Limonene oxide	0.5
68.99	Undecanoic acid, methyl ester	1.9
80.16	Cedrene	0.4
87.15	Phenylethyl salicylate	0.4
total		96.7

Table S21. Chemical constituents of patchuli essential oil

retention time (min)	constituent	area %
42.29	o-Cymene	11.1
43.04	Benzyl alcohol	2.8
48.35	Linalool	9.2
67.87	α-Patchoulene	0.9
68.66	α-Gurjunene	0.4
68.77	Isoledene	7.1
68.99	Undecanoic acid, methyl ester	2.0
69.18	Di-epi-α-cedrene	2.8
69.29	β-Caryophyllene	24.6
69.52	Cedrene	0.7
69.78	α-Guaiene	3.3
69.90	Thujopsene	3.7
70.50	Seychellene	1.9
70.81	Aromadendrene	3.2
70.93	α-Patchoulene	1.5
71.04	γ-Gurjunene	0.4
71.20	Azulene	1.3
71.80	Ledene	0.3
71.96	Azulene	0.7
72.17	α-Bulnesene	4.0
72.42	Benzene, 1-methyl-4-(1,2,2-trimethylcyclopentyl)-	1.3
75.47	Widdrol	0.5
75.58	Cedrol	3.2
75.89	Isoaromadendrene epoxide	0.3
76.79	Veridiflorol	0.5
77.46	Patchouli alcohol	8.0
total		95.7

Table S22. Chemical constituents of peppermint essential oil

retention time (min)	constituent	area %
27.04	α-Pinene	0.4
34.97	β-Pinene	0.7
42.31	o-Cymene	6.0
42.81	D-Limonene	6.8
48.37	Linalool	11.0
51.88	Isopulegol	0.8
52.47	Isomenthone	28.1
53.17	p-Menthone	11.2
53.52	Menthol	2.2
54.14	Levomenthol	18.4
54.32	Isopulegol	0.5
60.53	2-Cyclohexen-1-one, 3-methyl-6-(1-methylethyl)-	0.4
62.81	Menthyl acetate	1.6
69.00	Undecanoic acid, methyl ester	1.9
69.30	β-Caryophyllene	5.2
total		95.1

Table S23. Chemical constituents of pine essential oil

retention time (min)	constituent	area %
25.90	α-Pinene	43.9
28.40	Camphene	1.0
33.36	β-Pinene	7.6
36.00	α-Myrcene	2.2
39.40	3-Carene	8.0
41.45	o-Cymene	1.0
41.98	D-Limonene	5.9
42.10	α-Phellandrene	1.4
61.94	(-)-Bornyl acetate	0.6
68.48	Longifolene	0.4
68.60	Undecanoic acid, methyl ester	16.2
68.83	β-Caryophyllene	2.5
69.05	Undecanoic acid, 2-methyl	2.6
total		93.3

Table S24. Chemical constituents of rosemary essential oil

retention time (min)	constituent	area %
26.98	α-Pinene	15.9
29.63	Camphene	0.4
34.89	β-Pinene	9.0
40.41	3-Carene	1.8
42.27	o-Cymene	6.8
42.77	D-Limonene	7.9
43.05	Eucalyptol	30.3
45.40	γ-Terpinene	0.8
47.42	α-Terpinolene	1.1
51.78	Camphor	19.9
54.37	Terpinen-4-ol	0.3
68.98	Undecanoic acid, methyl ester	1.8
total		96.0

Table S25. Chemical constituents of sandal wood essential oil

retention time (min)	constituent	area %
42.96	Benzyl alcohol	14.1
68.87	Undecanoic acid, methyl ester	2.9
77.08	Norbornane	14.5
78.28	α-Santalol	5.8
80.71	Geranylgeraniol	3.9
81.02	Corymbolone	3.8
81.15	Eudesma-3,11-dien-2-one	11.1
81.27	2,6,11-Tridecatrien-10-ol, 2,6,10-trimethyl-	3.5
81.88	Longipinocarvone	4.4
82.21	Geranylgeraniol	3.4
82.43	1-Cyclohexene-1-butanal, à,2,6,6-tetramethyl-	5.0
82.61	Acetic acid, 1-[2-(2,2,6-trimethyl-bicyclo[4.1.0]hept-1-yl)-ethyl]-vinyl ester	8.9
82.88	9,17-Octadecadienal, (Z)-	2.1
total		83.4

Table S26. Chemical constituents of spearmint essential oil

retention time (min)	constituent	area %
26.65	*α*-Pinene	0.7
33.48	Sabinene	0.5
34.41	*β*-Pinene	0.9
37.08	Myrcene	1.6
38.34	3-Octanol	0.3
42.61	D-Limonene	21.2
42.82	Eucalyptol	2.1
53.89	Levomenthol	0.6
54.16	Terpinen-4-ol	0.6
55.60	Dihydrocarvone	1.8
59.75	(-)-Carvone	61.4
67.64	*α*-Bourbonene	0.8
68.87	Undecanoic acid, methyl ester	2.4
69.15	*β*-Caryophyllene	0.6
70.06	*trans*-*β*-Farnesene	0.4
71.39	Germacrene D	0.4
total		96.1

Table S27. Chemical constituents of tea tree essential oil

retention time (min)	constituent	area %
27.25	α-Pinene	4.6
41.72	α-Terpinene	3.7
42.60	o-Cymene	5.6
43.03	Limonene	1.2
43.35	Eucalyptol	4.8
45.64	γ-Terpinene	15.8
47.59	Terpinolene	6.8
54.91	Terpinen-4-ol	48.7
55.92	α-Terpineol	0.4
60.87	Piperitone	0.5
68.95	Undecanoic acid, methyl ester	1.7
total		93.9

Table S28. Chemical constituents of ylang ylang essential oil

retention time (min)	constituent	area %
41.08	4-Methylanisole	13.0
47.54	Methyl benzoate	5.8
47.98	Linalool	18.0
52.35	Benzyl acetate	19.9
54.74	Methyl salicylate	0.5
62.11	Anethole	0.4
66.78	Geranyl acetate	6.2
68.66	Undecanoic acid, methyl ester	3.0
68.93	β-Caryophyllene	4.8
69.58	Cinnamyl acetate	3.7
70.29	α-Caryophyllene	1.6
72.89	cis-α-Bisabolene	0.4
73.45	Nerolidol	0.4
79.60	Benzyl benzoate	3.8
83.34	Benzyl salicylate	14.6
total		95.9

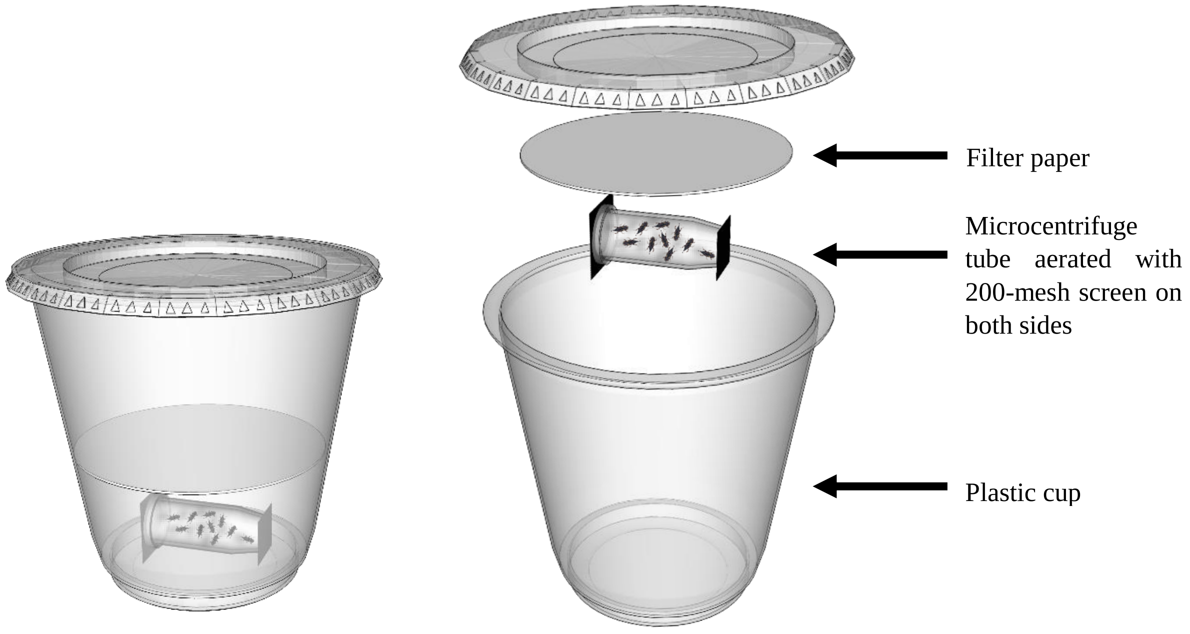

Fig. S1. Schematic diagram of fumigation assay arena.
